# Fluid overload modifies ambulatory blood pressure and pulse wave characteristics in hemodialysis patients undergoing targeted fluid management

**DOI:** 10.1093/ckj/sfag273

**Published:** 2026-08-12

**Authors:** Sebastian Mussnig, Luis Naar, Marieta Theodorakopoulou, Janosch Niknam Saeidi, Joachim Beige, David Keane, Daniel Schneditz, Pantelis Sarafidis, Siegfried Wassertheurer, Christopher C Mayer, Manfred Hecking

**Affiliations:** Department of Medicine III, Division for Nephrology and Dialysis, Medical University of Vienna, Vienna, Austria; Department of Medicine III, Division for Nephrology and Dialysis, Medical University of Vienna, Vienna, Austria; Center for Public Health, Department of Epidemiology, Medical University of Vienna, Vienna, Austria; Department of Medicine III, Division for Nephrology and Dialysis, Medical University of Vienna, Vienna, Austria; First Department of Nephrology, Hippokration Hospital, Aristotle University of Thessaloniki, Thessaloniki, Greece; Department of Medicine III, Division for Nephrology and Dialysis, Medical University of Vienna, Vienna, Austria; Kuratorium for Dialysis and Transplantation e.V. (KfH), Neu-Isenburg, Germany; Medical Clinic 2, Martin-Luther-University Halle/Wittenberg, Halle, Germany; CURAM, Center for Research in Medical Devices, University of Galway, Galway, Ireland; Otto Loewi Research Center for Vascular Biology, Immunology and Inflammation, Division of Physiology & Pathophysiology, Medical University of Graz, Graz, Austria; First Department of Nephrology, Hippokration Hospital, Aristotle University of Thessaloniki, Thessaloniki, Greece; Medical Signal Analysis, Center for Health & Bioresources, AIT Austrian Institute of Technology GmbH, Vienna, Austria; Medical Signal Analysis, Center for Health & Bioresources, AIT Austrian Institute of Technology GmbH, Vienna, Austria; Department of Medicine III, Division for Nephrology and Dialysis, Medical University of Vienna, Vienna, Austria; Kuratorium for Dialysis and Transplantation e.V. (KfH), Neu-Isenburg, Germany

**Keywords:** ambulatory blood pressure, fluid management, fluid overload, hemodialysis, pulse wave

## Abstract

**Background:**

Ambulatory blood pressure (BP) and pulse wave characteristics (PWCs) are stronger predictors of mortality than conventional pre-/post-dialytic BP in hemodialysis. While BP is recommended to be managed primarily by reducing fluid overload (FO), associations of FO with interdialytic BP/PWCs remain unknown.

**Methods:**

Bioimpedance-derived FO and interdialytic 44-h BP/PWCs were analyzed at baseline and follow-up of a quality improvement project involving targeted fluid management. Trended cosinor linear mixed-effects models assessed intra-patient associations of post-dialysis FO with interdialytic BP/PWCs.

**Results:**

We analyzed 120 visits (77/43 at baseline/follow-up) from 80 patients (70% male; mean age 68 ± 14 years; median dialysis vintage 38 [18–64] months). Post-dialysis FO decreased in baseline hypervolemic patients from 2.7 ± 1.3 L to 0.9 ± 1.7 L. Every 1 L increase in post-dialysis FO at the patient level was associated with increased peripheral BP (systolic: 4.54 mmHg, *P *< 0.001; diastolic: 2.55 mmHg, *P *< 0.001), peripheral resistance (0.03 s*mmHg/mL, *P *= 0.011), augmentation index (1.38%, *P *= 0.007), wave amplitudes (forward: 0.56 mmHg, *P *= 0.025; backward: 0.61 mmHg, *P *= 0.027), reservoir pressure peak (3.83 mmHg, *P *< 0.001), and pulse wave velocity (0.12 m/s, *P *= 0.007). Higher FO was associated with decreased interdialytic slopes of all BPs, peripheral resistance, backward wave amplitude, reservoir peak pressure, and pulse wave velocity. FO interacted with circadian rhythms of stroke volume, peripheral resistance, forward wave amplitude, and excess pressure peak.

**Conclusions:**

We showed that FO in hemodialysis is associated with increased interdialytic BP and PWCs on a patient level, and that FO blunts interdialytic BP/PWC trends. We further establish that physiological BP/PWC patterns may be associated with euvolemic fluid status, hence making the case for tailoring target weight using bioimpedance spectroscopy.

KEY LEARNING POINTS **What was known:**

- Among hemodialysis patients, elevated interdialytic ambulatory blood pressure and pulse wave characteristics, as well as fluid overload were strongly associated with mortality. However, the intra-patient association between changes in ambulatory blood pressure and fluid overload remains unknown.

**This study adds:**

- We found that post-dialysis fluid overload was strongly associated with interdialytic ambulatory blood pressure and pulse wave markers within-patient. Fluid overload blunted interdialytic blood pressure slopes and modified the physiological circadian patterns of hemodialysis patients.

**Potential impact:**

- These findings further establish that physiological patterns of ambulatory blood pressure and pulse wave characteristics are associated with euvolemic fluid status achieved by targeted fluid management, hence making the case for quantifying fluid overload and guiding target weight by bioimpedance spectroscopy when clinically plausible.

## INTRODUCTION

Interpretation of peri-dialytic blood pressure (BP) is historically enigmatic in hemodialysis patients. Early analyses from the 1990s [1] and later studies [2, 3] showed that elevated pre-dialysis BP was not associated with mortality. Compared to routine peri-dialytic BP, which is poorly reproducible and an imprecise estimate of the true interdialytic BP load [4], interdialytic 48-h BP monitoring carries more information on circadian patterns [5], BP variability [6], and interdialytic trends [7]. Elevated average 48-h BP [8], increased BP variability [9], the absence of nocturnal “dipping” [10–12], and pulse wave characteristics (PWCs) such as pulse wave velocity (PWV) were robust predictors of mortality [13, 14].

Current clinical recommendations suggest that detection and reduction of fluid overload (FO) in hemodialysis patients should be the primary lever for the management of BP [15]. Interventional trials, such as the “Dry-weight reduction in hypertensive hemodialysis patients (DRIP)” trial [16] or the “Lung Water by Ultrasound-Guided Treatment in Hemodialysis Patients (LUST)” ambulatory blood pressure measurement (ABPM) study demonstrated that lowering or fine-tuning dry weight significantly reduced both average interdialytic BP and PWCs [17, 18]. BP typically increases between dialysis treatments [19], and in an observational study, the slope of interdialytic BP steepened under targeted dry weight reduction [20].

However, changes in dry weight are an imprecise measure of fluid status. Body mass may undergo changes aside from variable fluid status [21], e.g., due to nutrition, exercise, or disease affecting lean and fat tissue mass. Fluid volumes can be quantified by bioimpedance spectroscopy, which uses low-to-high-frequency alternating currents [22] to estimate fluid volumes and body composition, including a dedicated FO compartment [23, 24]. This bioimpedance-derived FO was shown to be an independent predictor of mortality [25], and may be clinically more intuitive than interdialytic weight gain [26].

In a recent quality improvement project with the goal of improving fluid status and BP at an entire hemodialysis center [27], we showed that targeted fluid management based on bioimpedance-derived FO was able to significantly reduce pre- and post-dialysis BP. The aim of this sub-study was to analyze intra-patient associations of bioimpedance-derived FO with 44-h interdialytic BP/PWCs taken at two timepoints of our quality improvement intervention. Specifically, we hypothesized that achieving euvolemia may restore physiological interdialytic BP and PWC patterns.

## MATERIALS AND METHODS

### Population and ethics

This study is a sub-analysis of a quality improvement project conducted at the KfH dialysis center Weiden (Weiden in der Oberpfalz, Germany) between May 2024 and September 2024, which aimed to longitudinally assess and optimize fluid status, pruritus and depression in patients on maintenance hemodialysis. All patients of the KfH dialysis center Weiden treated with regular thrice-weekly in-center maintenance hemodialysis were included. Results of this project were previously published elsewhere [27, 28]. The quality improvement project was authorized by the Institutional Review Board of the KfH with a requirement to process pseudonymized data internally only. In addition, the requirement for an ethics review prior to the retrospective analysis and publication was waived by the Ethics Committee of the Bavarian State Medical Association after formal request.

### Study design

The design of the underlying quality improvement project was described in detail elsewhere [27]. Briefly, patients underwent three evaluation phases lasting 2–3 weeks during which we repeatedly measured FO, blood volume, in-center and interdialytic ambulatory BP/PWCs, and patient-reported outcomes (Fig. S1). The first two evaluation phases were each followed by a 2-week adjustment phase, in which treating physicians adapted treatment based on previous measurements to optimize fluid status. Data analyzed herein concern measurements which were conducted during the first (baseline, up to and including week 3) and last evaluation phase (follow-up, 8–10 weeks later).

### Procedures

#### Maintenance hemodialysis

During the baseline period, hemodialysis or hemodiafiltration were prescribed at the discretion of the treating physicians without knowledge of the quality improvement data and were later adapted based on quality improvement data up to and including the follow-up period.

#### Ambulatory BP monitoring

Patients were asked to undergo 52-h BP/PWC measurements across one interdialytic interval once at baseline (weeks 2–3) and once during follow-up (weeks 11–12) using the Mobil-O-Graph device (IEM GmbH, Aachen, Germany) [29–35]. Measurements were set to begin at the start of the second weekly dialysis treatment, they continued throughout the subsequent interdialytic interval and were terminated at the end of the third weekly dialysis treatment. Devices were programmed to measure BP/PWCs every 15 min during daytime (07:01–22:00) and every 30 min during nighttime (22:01–07:00). Additional PWCs (forward and backward wave amplitudes derived from wave separation analysis [36], reservoir and excess pressure peaks derived from reservoir theory [37, 38], and the S:D ratio derived from wave intensity analysis [39, 40]) were calculated at AIT Austrian Institute of Technology GmbH (Vienna, Austria) using MATLAB 2022b (The MathWorks Inc., Natick, MA, USA). The quality of estimated PWCs was assessed based on the percentage of usable pulse waves per reading (good: >80%, medium: 50%–80%, and poor: <50%).

#### Bioimpedance spectroscopy

Whole-body fluid status was scheduled to be measured either pre-dialysis or intra-dialysis at every dialysis treatment of the baseline and follow-up period using the Multiscan 5000 bioimpedance spectroscopy device (Bodystat Ltd, Douglas, Isle of Man). Intra-dialysis bioimpedance measurements were converted to pre-dialysis values by adding cumulative ultrafiltered fluid volumes at the time of the measurement during dialysis to extracellular fluid volume, total body fluid volume and FO [27]. Post-dialysis FO was computed by subtracting the cumulative ultrafiltration volume at the end of the respective treatment from pre-dialysis FO, assuming that ultrafiltered fluid only originated from extracellular fluid (and the FO compartment), and that intracellular fluid volumes were not affected by intradialytic fluid removal.

### Statistical analyses

#### Data preparation and exclusion criteria

When bioimpedance data were missing for a specific visit, post-dialysis FO was imputed from other treatments from the respective project phase. First, we averaged measurements from the same treatment session (e.g. first, second, or third of the week) within that specific phase (baseline or follow-up). If no session-specific data were available, FO was imputed using the average of all measurements from that entire phase. Only BP and PWC data recorded between dialysis treatments (∼44 h interval) were analyzed, and any records that coincided with the dialysis treatment, and cases with less than five BP/PWC datapoints were excluded.

#### Descriptive statistics and paired tests

Continuous variables were summarized as means (±standard deviation) or medians [quartile 1, quartile 3] depending on the distribution. Nominal and ordinal variables were described using counts and relative frequencies. Data were summarized either across patients or stratified by post-dialysis FO of each study visit, with strata for hypovolemia (<-1.1 L), euvolemia (-1.1 to +1.1 L), and hypervolemia (>1.1 L), referencing the 10th and 90th percentiles in healthy individuals. Note that each patient could contribute a maximum of two visits to summary tables and figures if valid measurements were recorded both at baseline and follow-up. Interdialytic averages of those patients who underwent BP/PWC measurements both at baseline and follow-up were compared between timepoints using paired *t*-tests.

BP/PWCs were scatter plotted against time overall, as well as stratified by post-dialysis FO per visit. Smooth curves (method “gam”) were added for each stratum. Measurements were additionally visualized as the average of intra-patient mean differences from the first measurement cycle (day 1 = day of the mid-week treatment) for each time of day (day: 07:01–23:00; night 23:01–07:00) with respective 95% confidence intervals (CIs).

#### Linear mixed-effects models

Associations of post-dialysis FO with BP/PWCs were investigated by trended cosinor [20] linear mixed-effects models, fitting BP/PWCs (continuous) as the dependent variable and post-dialysis FO (L, continuous) as the independent variable of interest. Specifically, we analyzed features indicative of arterial BP (central and peripheral BP), cardiac function (heart rate, stroke volume), arterial stiffness (PWV, augmentation index, and pulse pressure amplification), vasoconstriction (peripheral resistance, S:D ratio), wave reflection (forward and backward wave amplitudes), and reservoir theory (reservoir and excess pressures peaks).

**Figure 1:**
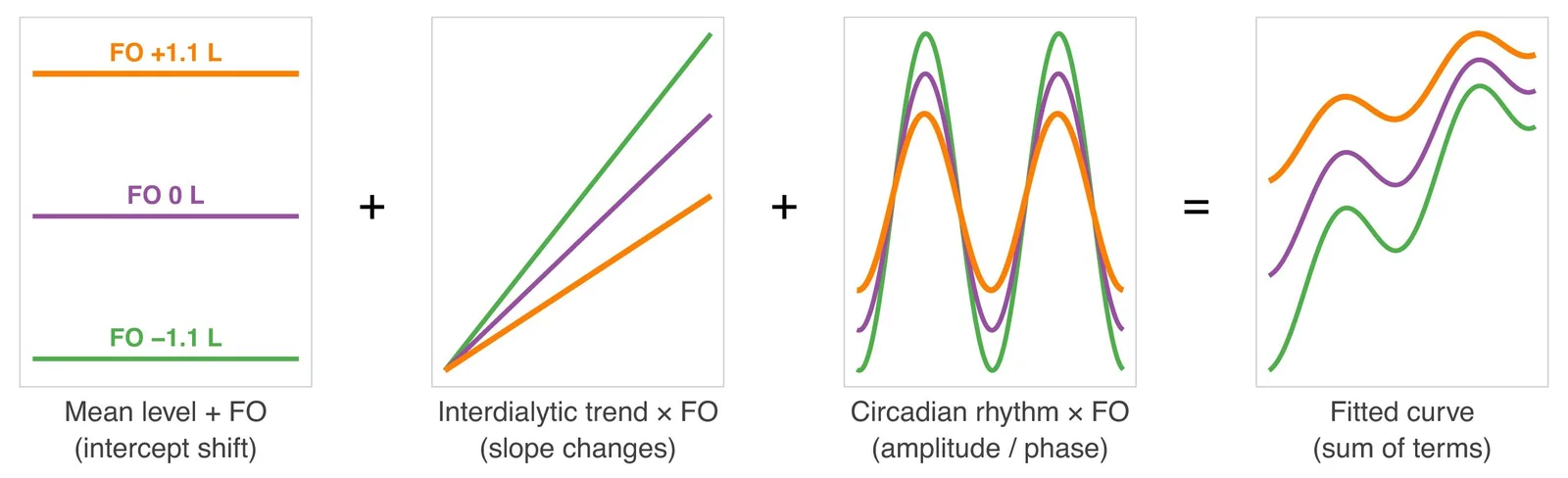
Overview of parameters and interactions of the trended cosinor model. Each panel shows one of the trended cosinor model’s terms and interactions of interest across the 44-h interdialytic interval, drawn at three levels of post-dialysis FO (−1.1, 0, and +1.1 L). From left to right: the mean level, comprising the intercept and the FO main effect, indicating BP or PWC values right after dialysis; the interdialytic trend over the interval, whose slope is progressively flattened by the FO × time interaction; the circadian rhythm, a 24-h cosine/sine oscillation whose amplitude (and, depending on the coefficients, phase) is altered by the FO × cosine and FO × sine interactions; and their sum, the fitted interdialytic curve. Abbreviation: FO, fluid overload.

Cosine and sine terms (i.e. $\cos ( {( {2 \times \pi \times \textit{hour}} )/24} )$ and $\sin ( {( {2 \times \pi \times \textit{hour}} )/24} )$) were fitted as fixed effects to capture circadian patterns of BP/PWCs. The time within the interdialytic interval (days, continuous) was fitted as a fixed effect to capture linear interdialytic trends of BP/PWCs. Interaction terms between post-dialysis FO and time within the interdialytic interval, and with cosine and sine terms were added to investigate modulating effects of FO on interdialytic trends and circadian rhythm. A schematic overview of these terms is shown in Fig. 1. Joint interactions of FO with sine and cosine terms were investigated by the Wald test.

Heart failure at baseline (binary), type 2 diabetes mellitus at baseline (binary), obstructive sleep apnea at baseline (binary), sex (male/female), age at baseline (years, continuous), and dialysis vintage at baseline (months, continuous), as well as the time-varying number of antihypertensive medications (ordinal) and dialysis shift (morning/noon/evening), were added as additional fixed effects to all models described above as potential confounders. Random intercepts were included for phase (baseline or follow-up) nested within patients. Models were fitted using *lme*, and a continuous autocorrelation structure of order 1 was specified. Model fits were investigated by conditional coefficients of determination (*R*^2^) and visual inspection of model residuals. In the primary analysis, we used PWCs independent of the quality of the estimates. We additionally conducted sensitivity analyses excluding PWCs with poor quality and excluding PWCs with poor and medium quality.

Predicted values of the trended cosinor linear mixed-effects models from the primary analysis were plotted at three levels of post-dialysis FO (−1.1, 0, and +1.1 L) to show associations of FO with intercept, interdialytic trend, amplitude, and phase. Population-averaged predictions were calculated from fitted models with confounders set at the sample means or sample medians. CIs were obtained via parametric bootstrap.

*P*-values across all baseline-to-follow-up comparisons, linear mixed-effects models of the primary analysis, and each sensitivity analysis were separately corrected to control the false discovery rate via the Benjamini–Hochberg procedure. Adjusted *P*-values were evaluated against the significance level of 0.05. All statistical analyses were carried out using R version 4.5.2 (2025-10-31) and RStudio for macOS.

## RESULTS

### Population characteristics and excluded data

Of 127 patients in the quality improvement project, ambulatory BP/PWC measurements were conducted in 86 (68%) during baseline visits and in 46 (36%) during follow-up visits (132 ambulatory BP/PWC readings in total). Twelve visits were excluded: five with only intradialytic recordings, five without FO, and two with fewer than 5 BP/PWC datapoints. Data from 80 patients (40 patients with data from both timepoints) and 120 visits (77 at baseline, 43 at follow-up) were included in the final analysis. Baseline population characteristics and comorbidities of this cohort are listed in Table 1. The population was predominantly male (*N* = 56, 70%), with a mean age of 68 (±14) years and median dialysis vintage of 38 [18, 64] months.

**Table 1:** Population characteristics.

Characteristic	*N*	Overall(80 patients)	Hypovolemia (19 patients)	Euvolemia(33 patients)	Hypervolemia (28 patients)
Age, years	80	68 (14)	65 (15)	68 (14)	71 (13)
Male sex	80	56 (70%)	12 (63%)	22 (67%)	22 (79%)
Dialysis modality	80				
Hemodiafiltration		6 (7.5%)	2 (11%)	0 (0%)	4 (14%)
Hemodialysis		74 (93%)	17 (89%)	33 (100%)	24 (86%)
Dialysis vintage, months	80	38 [18, 64]	43 [27, 74]	38 [12, 57]	38 [19, 68]
Residual urine output, mL/day	46	594 (515)	750 (523)	372 (339)	711 (592)
Cause of kidney failure	80				
Diabetes		14 (18%)	2 (11%)	4 (12%)	8 (29%)
Glomerular disease		24 (30%)	4 (21%)	13 (39%)	7 (25%)
Hypertension/vascular disease		19 (24%)	5 (26%)	6 (18%)	8 (29%)
Other		13 (16%)	5 (26%)	6 (18%)	2 (7.1%)
Unknown		10 (13%)	3 (16%)	4 (12%)	3 (11%)
History of kidney transplant	80	5 (6.3%)	2 (11%)	2 (6.1%)	1 (3.6%)
Obstructive sleep apnea syndrome	80	5 (6.3%)	0 (0%)	1 (3.0%)	4 (14%)
Type 2 diabetes mellitus	80	31 (39%)	4 (21%)	12 (36%)	15 (54%)
Heart failure	80	24 (30%)	2 (11%)	10 (30%)	12 (43%)
Arterial hypertension	80	63 (79%)	15 (79%)	26 (79%)	22 (79%)
Coronary artery disease	80	34 (43%)	7 (37%)	14 (42%)	13 (46%)
History of stroke	80	10 (13%)	4 (21%)	3 (9.1%)	3 (11%)
Albumin, g/dL	75	3.61 (0.42)	3.63 (0.34)	3.56 (0.51)	3.66 (0.35)
Hemoglobin, g/L	75	114 (15)	115 (13)	117 (12)	107 (19)
Antihypertensive drugs	80				
0		46 (58%)	15 (79%)	17 (52%)	14 (50%)
1		19 (24%)	3 (16%)	11 (33%)	5 (18%)
2		4 (5.0%)	0 (0%)	1 (3.0%)	3 (11%)
3		5 (6.3%)	0 (0%)	1 (3.0%)	4 (14%)
4 or more		6 (7.5%)	1 (5.3%)	3 (9.1%)	2 (7.1%)

The data are reported as mean (standard deviation), median [quartile 1, quartile 3], or frequency (percentage) overall and stratified by post-dialysis FO (hypovolemia: <−1.1 L; euvolemia: −1.1 to +1.1 L; and hypervolemia: >1.1 L) of the first visits per patient (i.e. at the baseline visit if the patient underwent interdialytic measurements at both timepoints, or at the follow-up visit otherwise).

### Clinical characteristics of FO strata

Clinical data from dialysis treatments preceding BP/PWC measurements are listed in Table 2. Post-dialysis FO averaged 0.1 (±2.1) L, was below −1.1 L in 35 visits (29%, hypovolemic), exceeded 1.1 L in 36 visits (30%, hypervolemic), and fell within −1.1 and +1.1 L in 49 visits (41%, euvolemic). Visits ending in hypervolemia exhibited lower interdialytic weight gains, lower ultrafiltration volumes, higher pre-dialysis FO, and higher pre- and post-dialysis systolic and diastolic BP compared to euvolemic and hypovolemic visits. Patients with post-dialysis hypervolemia at their first visit were more frequently men (79%) and were older [71 (±13) years] compared to euvolemic and hypovolemic patients (Table 1). Patients with valid measurements at both baseline and follow-up visits who were initially hypervolemic experienced a strong decrease in post-dialysis FO [*N* = 14, 2.7 (±1.3) L to 0.9 (±1.7) L], while changes were moderate for hypovolemic patients [*N* = 12, −2.3 (±0.6) L to −1.8 (±1.3) L], and fluid status did not change in euvolemic patients [*N* = 14, −0.3 (±0.6) L to −0.4 (±1.7) L]. The average number of antihypertensive medications increased from 0.9 (±1.4) to 1.2 (±1.5) per patient from baseline to follow-up.

**Table 2:** Characteristics of dialysis treatments preceding interdialytic BP and pulse wave measurements.

Characteristic	Visits	Overall (120 visits)	Hypovolemia (35 visits)	Euvolemia (49 visits)	Hypervolemia (36 visits)
Pre-dialysis body mass, kg	120	79.8 (18.4)	79.5 (21.6)	79.3 (18.8)	80.8 (14.5)
Post-dialysis body mass, kg	120	77.8 (18.0)	76.9 (20.7)	77.5 (18.7)	79.2 (14.3)
Target body mass, kg	120	77.4 (18.0)	76.4 (20.6)	77.1 (18.8)	78.8 (14.4)
Difference to target body mass, kg	120	0.4 (0.8)	0.5 (0.6)	0.4 (0.7)	0.3 (1.0)
Lean tissue mass, kg	120	41.2 (10.0)	40.4 (9.9)	41.4 (10.7)	41.5 (9.4)
Adipose tissue mass, kg	120	36.0 (17.5)	37.8 (19.2)	35.6 (18.3)	34.7 (14.7)
Interdialytic weight gain from last session, kg	120	2.0 (1.1)	2.6 (1.1)	1.9 (1.0)	1.6 (1.1)
Ultrafiltration volume, L	120	2.6 (1.2)	3.3 (1.1)	2.4 (1.1)	2.3 (1.2)
Dialysis duration, min	117	268 (19)	274 (19)	265 (18)	267 (20)
Ultrafiltration rate, mL/h/kg	117	7.6 (3.4)	9.2 (2.0)	7.4 (3.9)	6.4 (3.2)
Pre-dialysis fluid overload, L	120	2.7 (1.9)	1.3 (1.2)	2.4 (1.2)	4.6 (1.8)
Post-dialysis fluid overload, L	120	0.1 (2.1)	−2.3 (0.9)	-0.1 (0.6)	2.6 (1.2)
Pre-dialysis systolic blood pressure, mmHg	120	127 (21)	120 (19)	128 (24)	134 (16)
Pre-dialysis diastolic blood pressure, mmHg	120	72 (14)	70 (10)	71 (15)	75 (14)
Post-dialysis systolic blood pressure, mmHg	120	133 (26)	120 (23)	134 (27)	144 (21)
Post-dialysis diastolic blood pressure, mmHg	120	78 (15)	73 (12)	79 (16)	81 (14)

The data are reported as means (standard deviations) overall and stratified by post-dialysis FO of the respective visit (hypovolemia: <−1.1 L; euvolemia: −1.1 to +1.1 L; and hypervolemia: >1.1 L). Abbreviations: BP, blood pressure.

### Interdialytic BP and PWCs

**Figure 2:**
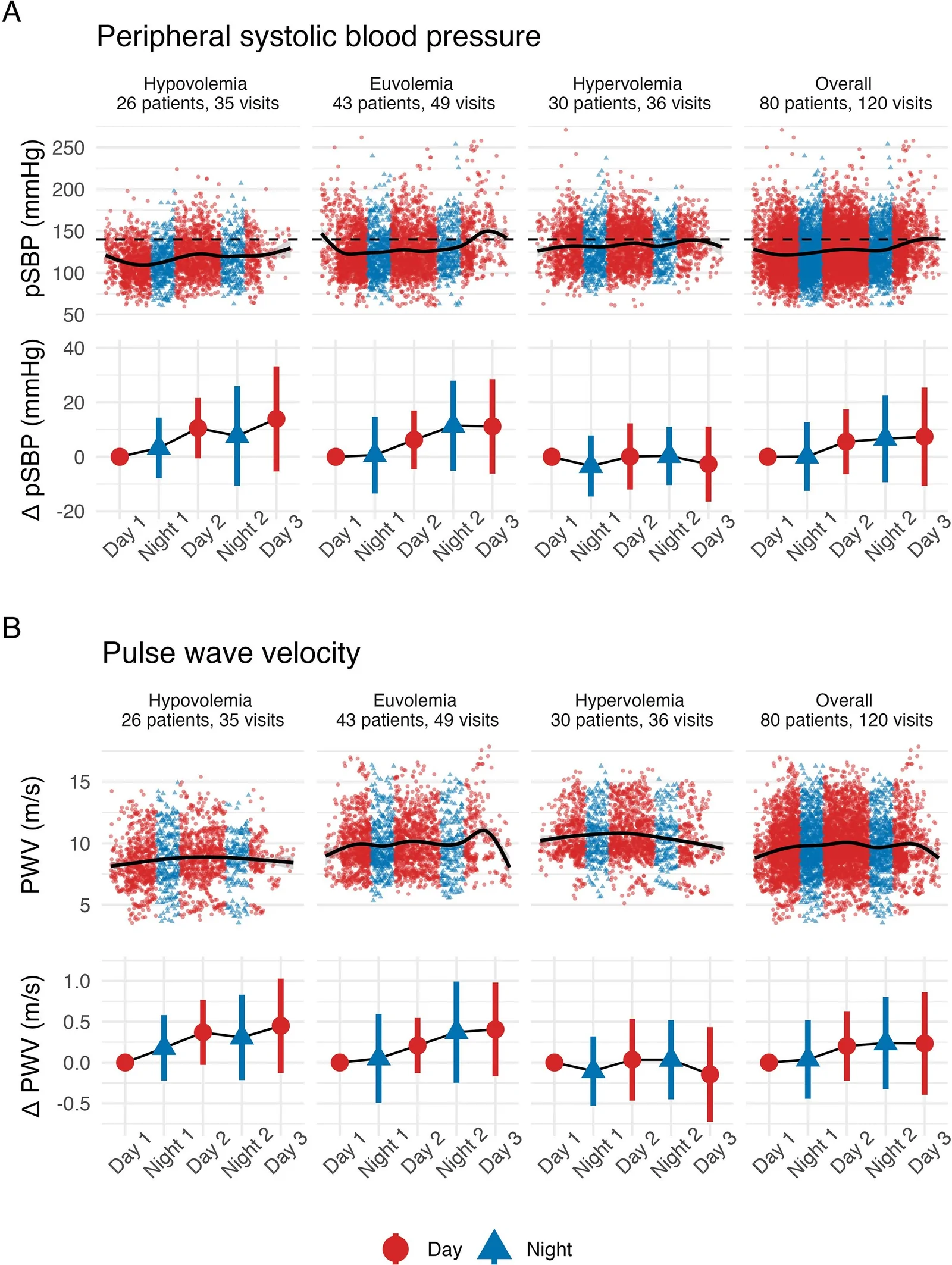
Interdialytic patterns of peripheral systolic blood pressure (pSBP) and pulse wave velocity (PWV). Panels A and B show interdialytic patterns of pSBP and PWV overall and stratified by post-dialysis fluid overload (FO, hypovolemia: <−1.1 L; euvolemia: −1.1 to +1.1 L; and hypervolemia: >1.1 L). The respective top row depicts values individually and with smooth curves (method “gam”). The respective bottom row depicts measurements as the visit-level average difference from the respective first measurement timepoint. Steepness indicates by how much pSBP/PWV changed from the first interdialytic measurement day. Circles (red) indicate measurements taken during the day (07:01–23:00) while triangles (blue) indicate measurements taken during the night (23:01–07:00). “Day 1” and “Day 3” indicate the days of the two dialysis treatments framing the interdialytic interval. Abbreviations: FO, fluid overload; pSBP, peripheral systolic blood pressure; PWV, pulse wave velocity.

Interdialytic BP/PWC data overall and stratified by post-dialysis FO are reported as means (of visits) in Table 3. Treatments ending in post-dialysis hypervolemia showed elevated BP/PWCs compared to euvolemic and hypovolemic visits across all variables under investigation, apart from pulse pressure amplification and heart rate (Table 3). Interdialytic trends of increasing BP, forward and backward wave amplitudes, augmentation index, peripheral resistance, and stroke volume were flattened among hypervolemic visits upon visual inspection (Fig. 2, Figs S2–S5). Intra-patient averages of all interdialytic BP variables, stroke volume, forward and backward wave amplitudes, and reservoir pressure peak significantly decreased from baseline to follow-up in patients with valid measurements at both timepoints, but pulse wave velocity did not change (Table S1).

**Table 3:** Averages of interdialytic BP and PWCs from baseline and follow-up visits.

Characteristic	Visits	Overall (120 visits)	Hypovolemia (35 visits)	Euvolemia (49 visits)	Hypervolemia (36 visits)
Peripheral systolic BP, mmHg	120	124.8 (21.2)	115.1 (17.6)	126.3 (23.0)	132.1 (18.6)
Peripheral diastolic BP, mmHg	120	72.5 (13.1)	68.6 (10.7)	73.2 (14.2)	75.3 (13.0)
Central systolic BP, mmHg	120	115.7 (20.5)	107.0 (17.5)	116.8 (21.8)	122.6 (18.7)
Central diastolic BP, mmHg	120	74.2 (13.7)	70.3 (11.8)	74.8 (14.5)	77.3 (13.7)
Heart rate, bpm	120	71 (12)	73 (14)	72 (12)	69 (10)
Stroke volume, mL	120	66 (10)	61 (9)	66 (11)	70 (8)
Pulse wave velocity, m/s	120	10.2 (2.3)	9.2 (2.3)	10.4 (2.3)	10.9 (2.1)
Augmentation index, %	120	28.1 (10.2)	25.7 (9.2)	27.5 (10.3)	31.2 (10.4)
Pulse pressure amplification, mmHg/mmHg	120	1.34 (0.13)	1.33 (0.08)	1.32 (0.11)	1.36 (0.19)
Peripheral resistance, s*mmHg/mL	120	1.29 (0.20)	1.26 (0.18)	1.28 (0.21)	1.32 (0.21)
S:D ratio, dimensionless	120	2.91 (1.02)	2.94 (1.14)	2.71 (0.80)	3.14 (1.13)
Forward pressure wave amplitude, mmHg	120	26.0 (7.4)	23.2 (6.6)	26.3 (7.7)	28.1 (7.0)
Backward pressure wave amplitude, mmHg	120	17.4 (5.6)	15.2 (5.2)	17.6 (6.0)	19.2 (4.6)
Reservoir pressure peak, mmHg	120	104.8 (18.5)	97.5 (16.2)	106.0 (19.7)	110.4 (17.1)
Excess pressure peak, mmHg	120	13.9 (3.5)	12.5 (2.7)	14.0 (3.7)	15.2 (3.3)

The data are reported as means of per-visit averages (both baseline and follow-up) overall and stratified by post-dialysis FO of the respective visit (hypovolemia: <−1.1 L; euvolemia: −1.1 to +1.1 L; and hypervolemia: >1.1 L). Abbreviations: BP, blood pressure.

### Associations of FO with BP and PWCs

**Figure 3:**
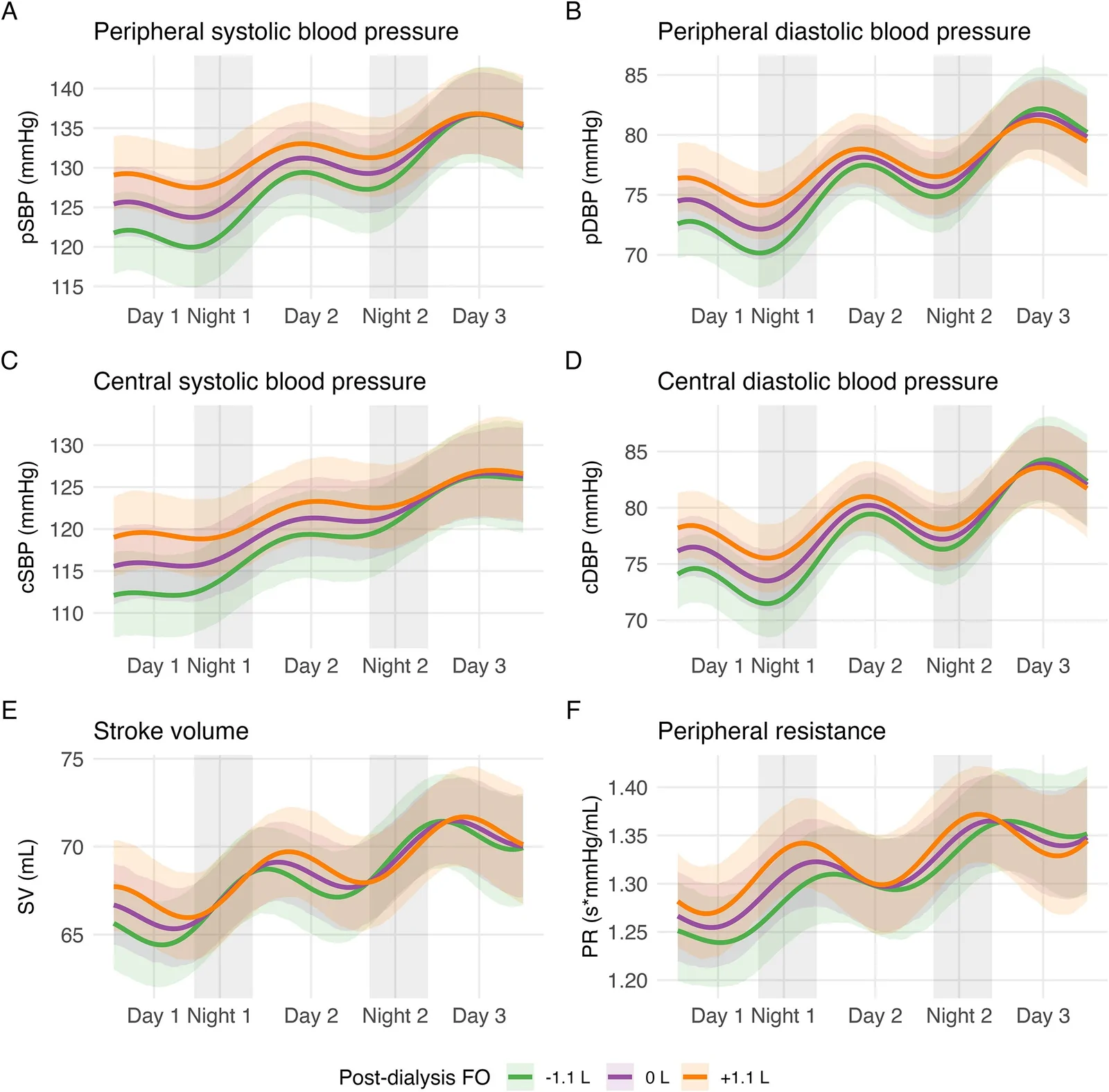
Predicted blood pressure (BP) and pulse wave characteristics (PWCs) at different levels of fluid overload (FO). Curves show predicted BPs and PWCs at three post-dialysis FO levels (−1.1, 0, and +1.1 L) derived from trended cosinor linear mixed-effects models. These curves visualize how different post-dialysis FO levels affect interdialytic intercepts, slopes, and circadian rhythms within a subject who is held otherwise constant for all other model confounders. Predictions were adjusted for confounders at sample means or medians. FO levels affected the start of the curve and interacted with the trend, circadian phase, and amplitude. Shaded areas show 95% CIs from parametric bootstrap. Shaded gray areas show nighttime hours (23:01–07:00). “Day 1” and “Day 3” indicate the days of the two dialysis treatments framing the interdialytic interval. Abbreviations: BP, blood pressure; cDBP and pDBP, central and peripheral diastolic blood pressure; cSBP and pSBP, central and peripheral systolic blood pressure; FO, fluid overload; PR, peripheral resistance; PWC, pulse wave characteristic; and SV, stroke volume.

**Figure 4:**
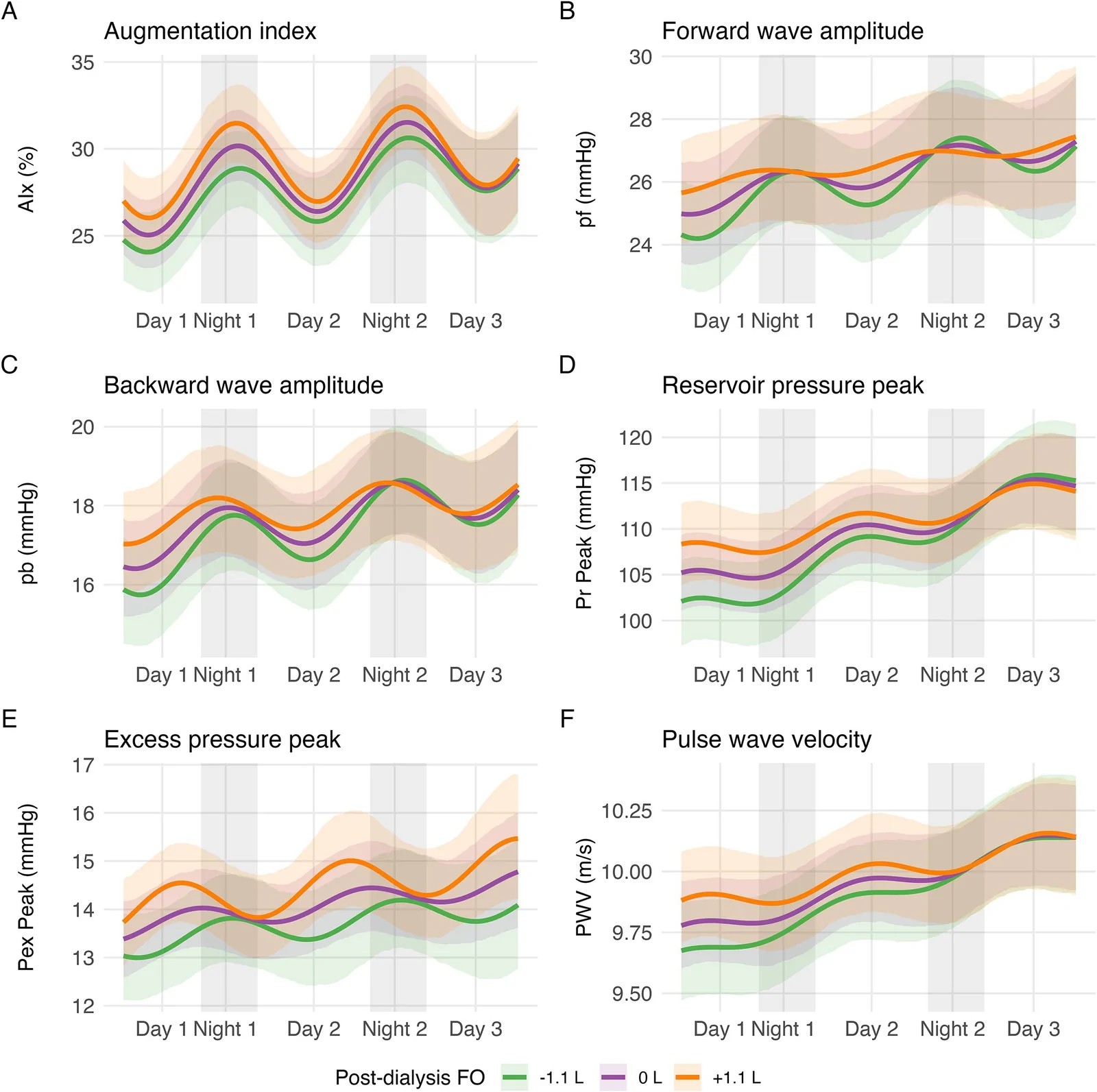
Predicted pulse wave characteristics (PWCs) at different levels of fluid overload (FO). Curves show predicted PWCs at three post-dialysis FO levels (−1.1, 0, and +1.1 L) derived from trended cosinor linear mixed-effects models. These curves visualize how different post-dialysis FO levels affect interdialytic intercepts, slopes, and circadian rhythms within a subject who is held otherwise constant for all other model confounders. Predictions were adjusted for confounders at sample means or medians. FO levels affected the start of the curve and interacted with the trend, circadian phase, and amplitude. Shaded areas show 95% CIs from parametric bootstrap. Shaded gray areas show nighttime hours (23:01–07:00). “Day 1” and “Day 3” indicate the days of the two dialysis treatments framing the interdialytic interval. Abbreviations: AIx, augmentation index; FO, fluid overload; pb, backward wave amplitude; Pex Peak, excess pressure peak; pf, forward wave amplitude; Pr Peak, reservoir pressure peak; PWC, pulse wave characteristic; and PWV, pulse wave velocity.

Effect estimates of trended cosinor linear mixed-effects models investigating intra-patient associations of post-dialysis FO with interdialytic BP/PWCs are listed in Table 4. For every 1 L increase in FO, we observed significant increases for all BPs, peripheral resistance, augmentation index, forward and backward wave amplitudes, reservoir pressure peak, and PWV. Negative interactions of FO with interdialytic trends of all interdialytic BPs, peripheral resistance, backward wave amplitude, reservoir pressure peak, and PWV indicated that FO reduced their interdialytic slope. Significant interactions with circadian patterns of stroke volume, peripheral resistance, forward wave amplitude, and excess pressure peak suggest that FO modified either their circadian amplitude or phase. Model predictions for three levels of FO (-1.1, 0, and +1.1 L) are shown in Figs 3 and 4, where the effects of FO on intercepts, trends, amplitudes, and phase are apparent. Estimates of trended cosinor linear mixed-effects models remained similar in sensitivity analyses compared to the primary analysis (Tables S2 and S3).

**Table 4:** Associations between post-dialysis FO and interdialytic BP and PWCs.

DV	*N*	Obs	β FO	P FO	β Trend × FO	P Trend × FO	P Circadian × FO	*R*²
Peripheral systolic BP, mmHg	80	6058	4.54 (2.59, 6.5)	<0.001	−1.6 (−2.27, −0.93)	<0.001	0.332	0.34
Peripheral diastolic BP, mmHg	80	6058	2.55 (1.38, 3.72)	<0.001	−1.05 (−1.47, −0.62)	<0.001	0.216	0.35
Central systolic BP, mmHg	80	3954	4.06 (2.12, 6)	<0.001	−1.48 (−2.24, −0.72)	<0.001	0.518	0.34
Central diastolic BP, mmHg	80	3954	2.62 (1.37, 3.88)	<0.001	−1.02 (−1.54, −0.51)	<0.001	0.460	0.36
Heart rate, bpm	80	6058	−0.19 (−1.21, 0.83)	0.725	0.15 (−0.16, 0.46)	0.431	0.163	0.46
Stroke volume, mL	80	3954	0.81 (−0.16, 1.78)	0.163	−0.33 (−0.74, 0.08)	0.173	0.027	0.18
Pulse wave velocity, m/s	80	3954	0.12 (0.04, 0.19)	0.007	−0.05 (−0.07, −0.02)	0.003	0.474	0.94
Augmentation index, %	80	3954	1.38 (0.54, 2.22)	0.007	−0.37 (−0.78, 0.04)	0.145	0.442	0.25
Pulse pressure amplification, mmHg/mmHg	80	5406	−0.01 (−0.02, 0.01)	0.464	0 (0, 0.01)	0.231	0.529	0.81
Peripheral resistance, s*mmHg/mL	80	3954	0.03 (0.01, 0.05)	0.011	−0.01 (−0.02, 0)	0.019	0.038	0.85
S:D ratio, dimensionless	80	5312	−0.02 (−0.12, 0.08)	0.725	0.03 (−0.01, 0.07)	0.182	0.149	0.43
Forward pressure wave amplitude, mmHg	80	3954	0.56 (0.15, 0.98)	0.025	−0.21 (−0.45, 0.03)	0.149	0.026	0.33
Backward pressure wave amplitude, mmHg	80	3954	0.61 (0.15, 1.08)	0.027	−0.23 (−0.42, −0.04)	0.033	0.093	0.36
Reservoir pressure peak, mmHg	80	3954	3.83 (2.07, 5.6)	<0.001	−1.59 (−2.31, −0.88)	<0.001	0.768	0.3
Excess pressure peak, mmHg	80	3954	0.28 (−0.07, 0.63)	0.173	0.04 (−0.15, 0.22)	0.725	0.007	0.21

Interdialytic BP and PWCs were modeled as dependent variables using trended cosinor linear mixed-effects models, adjusting for multiple confounders. “β FO” and “P FO” indicate the fixed effect (95% CI) and *P*-value of the independent variable of interest, post-dialysis FO, per 1 L change. “β Trend × FO” and “P Trend × FO” describe the interaction between post-dialysis FO and interdialytic trends, where a negative β would indicate that increased post-dialysis FO blunts interdialytic linear trends per day. “P Circadian × FO” indicates whether FO jointly interacted with sine and cosine terms of the model (Wald test), which would suggest an effect of FO on circadian patterns. Model fits were investigated by conditional coefficients of determination (*R*^2^). Random intercepts were included for phase (baseline or follow-up) nested within patients. Abbreviations: BP, blood pressure; DV, dependent variable; FO, fluid overload; and Obs, observations.

## DISCUSSION

In this study, we showed that post-dialysis FO was strongly associated with interdialytic ambulatory BP and PWCs, and that excess post-dialysis FO flattened patients’ interdialytic BP/PWC slopes and modified circadian patterns. Our results suggest that targeted fluid management among hypervolemic patients affects interdialytic BP/PWC slopes, independent of antihypertensive medications. Considering the strong association of elevated ambulatory BP with mortality among hemodialysis patients, targeting chronic FO may be a critical lever for mitigating deleterious cardiovascular outcomes in this vulnerable population.

Because vascular remodeling is unlikely to reverse within 8–10 weeks, the observed intra-patient associations of post-dialysis FO with BP/PWCs were likely driven by acute volume effects rather than structural changes. In hypervolemic states, excess interstitial fluid facilitates rapid plasma refilling during and after dialysis [41, 42], flattening interdialytic BP slopes by keeping the intravascular space persistently saturated. Gradual removal of excess whole-body FO may have tapered this refilling, so that BP trends instead depended on interdialytic fluid and sodium intake, yielding steeper BP/PWC slopes. This mechanism is supported by our finding that visits ending in hypovolemia were followed by higher interdialytic weight gains. Post-dialysis FO was also strongly associated with reservoir pressure, indicating that the Windkessel effect was not properly restored by insufficiently reducing plasma volume through ultrafiltration in states of post-dialysis hypervolemia. While post-dialysis FO showed statistically significant associations with wave reflection patterns, PWV, and peripheral resistance, their effect sizes were modest compared to the robust association with reservoir pressure. Our results suggest that the primary hemodynamic burden of hypervolemia may be mediated through impairment of the Windkessel function and sustained intravascular volume.

Preliminary studies relying on interdialytic weight gain as a surrogate for FO showed no associations with interdialytic BP and PWCs [5, 43] which is unsurprising, as interdialytic weight gain reflects short-term fluid deposits, and cannot be equated with chronic FO [26, 44]. In contrast, studies using direct, objective fluid assessment such as bioimpedance or anthropometric fluid estimates, consistently demonstrated strong associations between FO and higher average interdialytic BP [45–49]. The clearest evidence, however, came from interventional trials. In a sub-analysis of the DRIP study, Agarwal [50] analyzed interdialytic ambulatory BP in 100 hypertensive patients in the active arm (receiving targeted dry weight reductions throughout 8 weeks) compared to 50 hypertensive patients in the control arm, modeling interdialytic BP by trended cosinor analysis. In this trial, dry weight was reduced without any measure of objective FO, following a pre-defined algorithm of dry weight changes per treatment until symptoms occurred. Their observed changes in BP intercept were higher than those reported in our study, whereas changes to the BP slope were more pronounced in our cohort. In the LUST ABPM trial, in which a lung-ultrasound guided strategy was compared to standard of care for dry weight estimation, an average reduction of dry weight by 0.71 kg in the intervention group led to a decrease in 48-h ambulatory systolic and diastolic BP of 6.61 and 3.85 mmHg after 8 weeks, respectively, which is similar to changes observed among our patients. The change in lung B-lines was significantly correlated with the change in 48-h ambulatory systolic BP (*r* = 0.253, *P *= 0.033) and diastolic BP (*r* = 0.309, *P *= 0.01) [17]. The active arm also experienced a significant reduction of office and 48-h PWV, central pulse pressure and central systolic BP [18].

Compared to previous research efforts on this subject, our study has several strengths. We (i) measured FO directly instead of resorting to changes in body mass, clinically set dry weight or interdialytic weight gain, (ii) provoked an intra-patient change in fluid status through our quality improvement intervention, (iii) analyzed individual interdialytic BP/PWC measurements instead of intra-patient interdialytic averages, and (iv) applied a comprehensive modeling approach to compute associations between post-dialysis FO and interdialytic ambulatory BP/PWCs by adjusting for expected circadian and interdialytic dynamics and other confounders, and by accounting for inter-patient variability, thus uncovering true associations with FO on the patient level. The underlying project was limited by the quality improvement design, specifically the lack of a randomization process and a control arm, and the relatively small sample of patients who underwent measurements both at baseline and follow-up which may introduce the possibility of a selection bias. A further limitation is that within-patient change in FO was informed by the subset of patients measured at both timepoints. Precision for the effect-modification terms is therefore limited. Our estimation of post-dialysis FO assumed that ultrafiltered fluid only originated from the extracellular space as described in multiple studies [51–53], but some fluid may have originated from the intracellular compartment instead. As noted previously [27], the number of antihypertensive medications increased from baseline to follow-up in the entire quality improvement cohort, but the number of medications only slightly increased in the sub-population analyzed in this study. We addressed this shortcoming by introducing the number of antihypertensive medications to the models as a time-varying confounder; however, residual confounding remains possible.

We showed that FO in hemodialysis was associated with increased interdialytic BP and multiple PWCs on a patient level, and that FO blunted interdialytic BP/PWC trends. These findings further establish that physiological BP/PWC patterns may be associated with euvolemic fluid status, hence making the case for quantifying FO and guiding target weight by bioimpedance spectroscopy when clinically plausible.

## Supplementary Material

sfag273_Supplemental_File

## Data Availability

Consent for public disclosure of individual patient data was not obtained at the time of data collection. Data can therefore not be shared on a public repository.
