# Supplementary material for: Fluid overload modifies ambulatory blood pressure and pulse wave characteristics in hemodialysis patients undergoing targeted fluid management
Source: Clin Kidney J. 2026 Aug 12;19(9):sfag273. doi: 10.1093/ckj/sfag273 (PMC13542727; doi:10.1093/ckj/sfag273)
Supplement: Supplementary file 1 [file sfag273_supplemental_file.docx]

**Supplemental Materials to:**

**Associations of fluid overload with ambulatory blood pressure and pulse wave characteristics in hemodialysis patients undergoing targeted fluid management**

Sebastian Mussnig, Luis Naar, Marieta Theodorakopoulou, Janosch Niknam, Joachim Beige, David Keane, Daniel Schneditz, Pantelis Sarafidis, Siegfried Wassertheurer, Christopher C Mayer, Manfred Hecking

Correspondence to:

Manfred Hecking (manfred.hecking@meduniwien.ac.at)

Medical University of Vienna
Department of Medicine III, Division for Nephrology and Dialysis

Währinger Gürtel 18-20
1090 Vienna, Austria

**Supplemental Methods**

*Multiple testing and software*

P-values across all baseline-to-follow-up comparisons, linear mixed-effects models of the primary analysis, and each sensitivity analysis were separately corrected to control the false discovery rate via the Benjamini-Hochberg procedure. Adjusted P-values were evaluated against the significance level of 0.05. All statistical analyses were carried out using R version 4.5.2 (2025-10-31) and RStudio for macOS.

Table S1: Baseline and follow-up measures of interdialytic blood pressure and pulse wave characteristics

| **Characteristic** | **N** | **Overall (80 visits)** | **Baseline (40 visits)** | **Follow-up (40 visits)** | **P-value** |
| --- | --- | --- | --- | --- | --- |
| Peripheral systolic BP, mmHg | 80 | 122.5 (20.4) | 126.4 (20.1) | 118.5 (20.3) | 0.004 |
| Peripheral diastolic BP, mmHg | 80 | 71.7 (12.8) | 74.2 (12.8) | 69.2 (12.5) | 0.003 |
| Central systolic BP, mmHg | 80 | 114.1 (20.1) | 118.0 (20.1) | 110.2 (19.6) | 0.004 |
| Central diastolic BP, mmHg | 80 | 73.6 (13.3) | 76.3 (13.3) | 71.0 (12.8) | 0.004 |
| Heart rate, bpm | 80 | 71 (12) | 71 (10) | 72 (14) | 0.4 |
| Stroke volume, mL | 80 | 65 (10) | 67 (8) | 64 (11) | 0.033 |
| Pulse wave velocity, m/s | 80 | 10.1 (2.1) | 10.2 (2.2) | 10.1 (2.1) | 0.3 |
| Augmentation index, % | 80 | 28.1 (10.5) | 28.9 (10.3) | 27.3 (10.8) | 0.12 |
| Pulse pressure amplification, mmHg/mmHg | 80 | 1.33 (0.12) | 1.33 (0.10) | 1.34 (0.13) | 0.2 |
| Peripheral resistance, s*mmHg/mL | 80 | 1.29 (0.21) | 1.31 (0.22) | 1.27 (0.21) | 0.075 |
| S:D ratio, dimensionless | 80 | 2.88 (0.97) | 2.88 (1.07) | 2.87 (0.87) | >0.9 |
| Forward pressure wave amplitude, mmHg | 80 | 25.4 (7.6) | 26.2 (8.0) | 24.7 (7.2) | 0.038 |
| Backward pressure wave amplitude, mmHg | 80 | 16.9 (5.6) | 17.4 (5.5) | 16.3 (5.7) | 0.030 |
| Reservoir pressure peak, mmHg | 80 | 103.5 (18.4) | 107.1 (18.5) | 99.9 (17.8) | 0.003 |
| Excess pressure peak, mmHg | 80 | 13.6 (3.2) | 13.9 (3.1) | 13.3 (3.2) | 0.2 |

Legend to S1: The data are reported as means (of case averages) (standard deviation of case averages) overall and stratified by project phase (“Baseline” and “Follow-up”). Fluid management was systematically adapted between these two phases with the express goal of lowering bioimpedance-derived fluid overload. Results from paired t-tests comparing both timepoints are indicated by P-values, which were corrected to control the false discovery rate via the Benjamini-Hochberg procedure, and which were evaluated against the significance level of 0.05. Note that this analysis only included patients who underwent ambulatory blood pressure and pulse wave measurements at both timepoints. Abbreviations: BP, blood pressure; C, case.

Table S2: Associations between post-dialysis fluid overload and interdialytic blood pressure and pulse wave characteristics using only observations with good or medium quality

| **DV** | **N** | **Obs** | **β FO** | **P FO** | **β Trend × FO** | **P Trend × FO** | **P Circadian × FO** | **R²** |
| --- | --- | --- | --- | --- | --- | --- | --- | --- |
| Peripheral systolic BP, mmHg | 79 | 3438 | 4.39 (2.3, 6.48) | <0.001 | -1.58 (-2.41, -0.75) | <0.001 | 0.670 | 0.33 |
| Peripheral diastolic BP, mmHg | 79 | 3438 | 2.55 (1.32, 3.79) | <0.001 | -1.16 (-1.69, -0.64) | <0.001 | 0.662 | 0.35 |
| Central systolic BP, mmHg | 79 | 3438 | 4.15 (2.19, 6.11) | <0.001 | -1.6 (-2.41, -0.79) | <0.001 | 0.475 | 0.33 |
| Central diastolic BP, mmHg | 79 | 3438 | 2.56 (1.31, 3.82) | <0.001 | -1.15 (-1.69, -0.61) | <0.001 | 0.662 | 0.35 |
| Heart rate, bpm | 79 | 3438 | -0.2 (-1.28, 0.88) | 0.745 | 0.15 (-0.25, 0.55) | 0.544 | 0.143 | 0.44 |
| Stroke volume, mL | 79 | 3438 | 0.68 (-0.32, 1.68) | 0.240 | -0.33 (-0.78, 0.11) | 0.199 | 0.010 | 0.19 |
| Pulse wave velocity, m/s | 79 | 3438 | 0.12 (0.05, 0.2) | 0.006 | -0.05 (-0.08, -0.02) | 0.002 | 0.356 | 0.94 |
| Augmentation index, % | 79 | 3438 | 1.63 (0.79, 2.47) | 0.001 | -0.41 (-0.84, 0.02) | 0.100 | 0.412 | 0.23 |
| Pulse pressure amplification, mmHg/mmHg | 79 | 3438 | -0.01 (-0.02, 0) | 0.034 | 0.01 (0, 0.01) | 0.056 | 0.348 | 0.75 |
| Peripheral resistance, s*mmHg/mL | 79 | 3438 | 0.03 (0.01, 0.05) | 0.006 | -0.01 (-0.02, 0) | 0.014 | 0.049 | 0.85 |
| S:D ratio, dimensionless | 79 | 3436 | 0.08 (-0.01, 0.18) | 0.141 | 0.03 (0, 0.07) | 0.125 | 0.742 | 0.64 |
| Forward pressure wave amplitude, mmHg | 79 | 3438 | 0.89 (0.27, 1.51) | 0.015 | -0.25 (-0.5, 0.01) | 0.096 | 0.030 | 0.36 |
| Backward pressure wave amplitude, mmHg | 79 | 3438 | 0.73 (0.27, 1.19) | 0.008 | -0.23 (-0.43, -0.03) | 0.047 | 0.060 | 0.36 |
| Reservoir pressure peak, mmHg | 79 | 3438 | 3.93 (2.14, 5.72) | <0.001 | -1.66 (-2.43, -0.89) | <0.001 | 0.827 | 0.29 |
| Excess pressure peak, mmHg | 79 | 3438 | 0.35 (-0.01, 0.71) | 0.096 | 0 (-0.21, 0.2) | 0.982 | 0.020 | 0.19 |

Legend to Table S2: Only observations with >50% availability of pulse waves per individual BP/PWC measurements were included in this sensitivity analysis. Interdialytic blood pressure and pulse wave characteristics were modelled as dependent variables using trended cosinor linear mixed-effects models, adjusting for multiple confounders. “β FO” and “P FO” indicate the fixed effect (95% confidence interval [CI]) and p-value of the independent variable of interest, post-dialysis fluid overload (FO), per 1 L change. “β Trend × FO” and “P Trend × FO” describe the interaction between post-dialysis FO and interdialytic trends, where a negative β would indicate that increased post-dialysis FO blunts interdialytic linear trends per day. “P Circadian × FO” indicates whether FO jointly interacted with sine and cosine terms of the model (Wald test), which would suggest an effect of FO on circadian patterns. Model fits were investigated by conditional coefficients of determination (R^2^). Random intercepts were included for phase (baseline or follow-up) nested within patients. Abbreviations: BP, blood pressure; DV, dependent variable; FO, fluid overload; Obs, observations.

Table S3: Associations between post-dialysis fluid overload and interdialytic blood pressure and pulse wave characteristics using only observations with good quality

| **DV** | **N** | **Obs** | **β FO** | **P FO** | **β Trend × FO** | **P Trend × FO** | **P Circadian × FO** | **R²** |
| --- | --- | --- | --- | --- | --- | --- | --- | --- |
| Peripheral systolic BP, mmHg | 74 | 2078 | 4.75 (2.45, 7.05) | 0.001 | -1.86 (-2.95, -0.77) | 0.004 | 0.945 | 0.33 |
| Peripheral diastolic BP, mmHg | 74 | 2078 | 2.96 (1.65, 4.27) | <0.001 | -1.57 (-2.23, -0.91) | <0.001 | 0.440 | 0.35 |
| Central systolic BP, mmHg | 74 | 2078 | 4.6 (2.44, 6.75) | <0.001 | -1.95 (-3, -0.91) | 0.001 | 0.885 | 0.33 |
| Central diastolic BP, mmHg | 74 | 2078 | 2.96 (1.62, 4.3) | <0.001 | -1.54 (-2.21, -0.87) | <0.001 | 0.485 | 0.35 |
| Heart rate, bpm | 74 | 2078 | 0.11 (-1.02, 1.24) | 0.903 | 0.1 (-0.36, 0.55) | 0.811 | 0.038 | 0.43 |
| Stroke volume, mL | 74 | 2078 | 0.54 (-0.39, 1.47) | 0.359 | -0.21 (-0.79, 0.37) | 0.596 | 0.029 | 0.21 |
| Pulse wave velocity, m/s | 74 | 2078 | 0.13 (0.05, 0.21) | 0.008 | -0.06 (-0.1, -0.02) | 0.006 | 0.811 | 0.94 |
| Augmentation index, % | 74 | 2078 | 1.96 (0.87, 3.05) | 0.004 | -0.78 (-1.39, -0.17) | 0.029 | 0.464 | 0.28 |
| Pulse pressure amplification, mmHg/mmHg | 74 | 2078 | -0.01 (-0.02, 0) | 0.010 | 0.01 (0, 0.02) | 0.077 | 0.883 | 0.12 |
| Peripheral resistance, s*mmHg/mL | 74 | 2078 | 0.04 (0.01, 0.06) | 0.008 | -0.02 (-0.03, -0.01) | 0.010 | 0.254 | 0.86 |
| S:D ratio, dimensionless | 74 | 2076 | 0.11 (0.03, 0.19) | 0.029 | 0.05 (-0.01, 0.1) | 0.131 | 0.883 | 0.57 |
| Forward pressure wave amplitude, mmHg | 74 | 2078 | 0.83 (0.13, 1.53) | 0.044 | -0.22 (-0.55, 0.11) | 0.274 | 0.123 | 0.37 |
| Backward pressure wave amplitude, mmHg | 74 | 2078 | 0.76 (0.24, 1.28) | 0.014 | -0.25 (-0.51, 0) | 0.095 | 0.198 | 0.37 |
| Reservoir pressure peak, mmHg | 74 | 2078 | 4.54 (2.56, 6.52) | <0.001 | -2.02 (-3.01, -1.02) | <0.001 | 0.903 | 0.31 |
| Excess pressure peak, mmHg | 74 | 2078 | 0.18 (-0.23, 0.59) | 0.485 | 0.02 (-0.24, 0.28) | 0.903 | 0.123 | 0.22 |

Legend to Table S3: Only observations with >80% availability of pulse waves per individual BP/PWC measurements were included in this sensitivity analysis. Interdialytic blood pressure and pulse wave characteristics were modelled as dependent variables using trended cosinor linear mixed-effects models, adjusting for multiple confounders. “β FO” and “P FO” indicate the fixed effect (95% confidence interval [CI]) and p-value of the independent variable of interest, post-dialysis fluid overload (FO), per 1 L change. “β Trend × FO” and “P Trend × FO” describe the interaction between post-dialysis FO and interdialytic trends, where a negative β would indicate that increased post-dialysis FO blunts interdialytic linear trends per day. “P Circadian × FO” indicates whether FO jointly interacted with sine and cosine terms of the model (Wald test), which would suggest an effect of FO on circadian patterns. Model fits were investigated by conditional coefficients of determination (R^2^). Random intercepts were included for phase (baseline or follow-up) nested within patients. Abbreviations: BP, blood pressure; DV, dependent variable; FO, fluid overload; Obs, observations.

Figure S1: Phases of the quality improvement project

Legend to Figure S1: The quality improvement project lasted 14 weeks. Patients and nurses reported how they perceived the patient’s fluid status during Check-In (week 1) and Check-Out (weeks 13-14). Electronic patient-reported outcome measures (ePROMs) were captured at each dialysis treatment between weeks 2-12. Bioimpedance-spectroscopy-(BIS)-derived fluid overload and body composition, and absolute blood volume (ABV) estimations were acquired at each treatment during Evaluation phases. The RECOVA symptom score was recorded once per Evaluation and Adjustment phase. Interdialytic ambulatory 48-h measurements of blood pressure and pulse wave characteristics (48h BP/PWC) were recorded once during Evaluation 1 and Evaluation 3. Data were algorithmically summarized prior to each Adjustment phase to single-page synopses. Subsequent treatment adjustments were guided by these summaries. Variables with superscript (1) were recorded once per phase, while variables with superscript (2) were recorded during each treatment of the respective phase. Abbreviations: ABV, absolute blood volume estimated by the dialysate-bolus method; BIS, bioimpedance spectroscopy; BP, blood pressure; ePROMs, electronically-captured patient-reported outcome measures; PWC, pulse wave characteristic.

Figure S2: Interdialytic patterns of central and peripheral blood pressures

Legend to Figure S2: Panels A, B and C show interdialytic patterns of central systolic blood pressure (cSBP), central diastolic blood pressure (cDBP) and peripheral diastolic blood pressure (pDBP) overall and stratified by post-dialysis fluid overload (hypovolemia: <-1.1 L; euvolemia: -1.1 to +1.1 L; hypervolemia: >1.1 L). The respective top row depicts values individually and with smooth curves (method “gam”). The respective bottom row depicts measurements as the case-level average difference from the respective first measurement timepoint. Steepness indicates by how much cSBP/cDBP/pDBP changed from the first interdialytic measurement day. Spheres (red) indicate measurements taken during the day (07:01 to 23:00) while triangles (blue) indicate measurements taken during the night (23:01 to 07:00). “Day 1” and “Day 3” indicate the days of the two dialysis treatments framing the interdialytic interval. Abbreviations: cSBP, central systolic blood pressure; cDBP and pDBP, central and peripheral diastolic blood pressure.

Figure S3: Interdialytic patterns of stroke volume, peripheral resistance and augmentation index

Legend to Figure S3: Panels A, B and C show interdialytic patterns of stroke volume (SV), peripheral resistance (PR) and augmentation index (AIx) overall and stratified by post-dialysis fluid overload (hypovolemia: <-1.1 L; euvolemia: -1.1 to +1.1 L; hypervolemia: >1.1 L). The respective top row depicts values individually and with smooth curves (method “gam”). The respective bottom row depicts measurements as the case-level average difference from the respective first measurement timepoint. Steepness indicates by how much SV/PR/AIx changed from the first interdialytic measurement day. Spheres (red) indicate measurements taken during the day (07:01 to 23:00) while triangles (blue) indicate measurements taken during the night (23:01 to 07:00). “Day 1” and “Day 3” indicate the days of the two dialysis treatments framing the interdialytic interval. Abbreviations: AIx, augmentation index; PR, peripheral resistance; SV, stroke volume.

Figure S4: Interdialytic patterns of forward and backward wave amplitudes

Legend to Figure S4: Panels A and B show interdialytic patterns of forward (pf) and backward wave amplitudes (pb) overall and stratified by post-dialysis fluid overload (hypovolemia: <-1.1 L; euvolemia: -1.1 to +1.1 L; hypervolemia: >1.1 L). The respective top row depicts values individually and with smooth curves (method “gam”). The respective bottom row depicts measurements as the case-level average difference from the respective first measurement timepoint. Steepness indicates by how much pf/pb changed from the first interdialytic measurement day. Spheres (red) indicate measurements taken during the day (07:01 to 23:00) while triangles (blue) indicate measurements taken during the night (23:01 to 07:00). “Day 1” and “Day 3” indicate the days of the two dialysis treatments framing the interdialytic interval. Abbreviations: pf, forward wave amplitude; pb, backward wave amplitude.

Figure S5: Interdialytic patterns of reservoir and excess pressure peaks

Legend to Figure S5: Panels A and B show interdialytic patterns of reservoir (Pr Peak) and excess pressure peaks (Pex Peak) overall and stratified by post-dialysis fluid overload (hypovolemia: <-1.1 L; euvolemia: -1.1 to +1.1 L; hypervolemia: >1.1 L). The respective top row depicts values individually and with smooth curves (method “gam”). The respective bottom row depicts measurements as the case-level average difference from the respective first measurement timepoint. Steepness indicates by how much Pr Peak/Pex Peak changed from the first interdialytic measurement day. Spheres (red) indicate measurements taken during the day (07:01 to 23:00) while triangles (blue) indicate measurements taken during the night (23:01 to 07:00). “Day 1” and “Day 3” indicate the days of the two dialysis treatments framing the interdialytic interval. Abbreviations: Pex Peak, excess pressure peak; PR Peak, reservoir pressure peak.
